# Salbutamol Worsens the Autonomic Nervous System Dysfunction of Children With Sickle Cell Disease

**DOI:** 10.3389/fphys.2020.00031

**Published:** 2020-02-26

**Authors:** Plamen Bokov, Houmam El Jurdi, Isabelle Denjoy, Claudine Peiffer, Noria Medjahdi, Laurent Holvoet, Malika Benkerrou, Christophe Delclaux

**Affiliations:** ^1^Service de Physiologie Pédiatrique, AP-HP, Hôpital Robert Debré, Paris, France; ^2^UMR 1141, Equipe NeoPhen, INSERM co-tutelle, Université de Paris, Paris, France; ^3^Service d’Hématologie Pédiatrique, AP-HP, Hôpital Robert Debré, Paris, France

**Keywords:** sickle cell disease, asthma, salbutamol, heart rate variability, sympathetic activity, vagal activity, vaso-occlusive event

## Abstract

**Background:**

Sickle cell disease (SCD) patients with asthma have an increased rate of vaso-occlusive crisis (VOC) and acute chest syndrome (ACS) episodes when compared to those without asthma. We hypothesized that either asthma diagnosis or bronchodilator treatment might aggravate SCD via their modulating effect on the autonomic nervous system (ANS).

**Methods:**

Cross-sectional evaluation of heart rate variability (HRV) during pulmonary function tests, including salbutamol administration, in children with SCD receiving asthma treatment or not when compared to asthmatic children without SCD matched for ethnicity.

**Results:**

SCD children with asthma (*n* = 30, median age of 12.9 years old) were characterized by a reduced FEV_1_/FVC ratio, an increased bronchodilator response, and a greater incidence of VOC and ACS when compared to SCD children without asthma (*n* = 30, 12.7 years). Children with asthma without SCD (*n* = 29, 11.4 years) were characterized by a higher exhaled NO fraction than SCD children. SCD children when compared to non-SCD children showed reduced HRV [total power, low (LF) and high (HF, vagal tone) frequencies], which was further worsened by salbutamol administration in all the groups: reduction in total power and HF with an increase in LF/HF ratio. After salbutamol, the LF/HF ratio of the SCD children was higher than that of the non-SCD children. The two groups of SCD children were similar, suggesting that asthma diagnosis *per se* did not modify ANS functions.

**Conclusion:**

SCD children are characterized by impaired parasympathetic control and sympathetic overactivity that is worsened by salbutamol administration.

**Clinical Trial Registration:**

www.ClinicalTrials.gov, identifier NCT04062409.

## Introduction

Asthma diagnosis in sickle cell disease (SCD) patients is associated with worse prognosis, i.e., increased morbidity and mortality (Glassberg et al., 2014). As stated by Mehari and Klings, one of the difficulties in diagnosing asthma in SCD stems from the overlap of clinical features, which may be solely due to SCD and those of asthma in the non-SCD population (Mehari and Klings, 2016). The use of beta_2_-agonists in children with SCD and asthma confounds interpretation of whether asthma or asthma treatment is associated with worse SCD as measured by incident acute chest syndrome (ACS) and vaso-occlusive crisis (VOC). For instance, in the study by Glassberg et al., including 74 SCD children, painful episodes with adequate documentation of respiratory symptoms were more frequently preceded by respiratory symptoms in the group with asthma when compared with those without asthma (Glassberg et al., 2006). One may hypothesize that these respiratory symptoms led to the intensification of bronchodilator intake that may further precipitate VOC. Similarly, in the study by Sylvester et al. (2007) more of the children who had an ACS compared to those who did not were taking anti-asthma medication.

Our working hypothesis is that beta_2_-agonists may aggravate SCD via their modulating effect on the autonomic nervous system (ANS). The few studies that have investigated ANS functions in patients with SCD have reported alterations such as decreased parasympathetic activity and sympathetic activity predominance in steady-state condition (Romero Mestre et al., 1997; Pearson et al., 2005). Furthermore, ANS dysfunction seems to be a marker of severity in SCD (Nebor et al., 2011). Beta_2_-agonists may further modify ANS balance. Acute salbutamol administration increases sympathetic activity in both healthy and asthmatic subjects (Eryonucu et al., 2001; Edgell et al., 2016). As a consequence, one may hypothesize that beta_2_-agonists may aggravate ANS dysfunction of SCD that is already characterized by enhanced sympathetic activity when compared to its effect in asthmatic patients who exhibit on the opposite an enhanced parasympathetic activity (Garrard et al., 1992).

Thus, our objectives were to assess ANS functions based on heart rate variability (HRV) in patients with SCD receiving asthma treatment or not, when compared to asthmatic children without SCD, who were matched for ethnicity since its influences HRV (Hill et al., 2015), and to assess the effect of salbutamol administration on ANS in these three groups of children.

## Materials and Methods

This cross-sectional case (SCD)–control (non-SCD) study complied with STROBE guidelines.

### Patients

Subjects of Sub-Saharan African or Caribbean ethnicity who were 8 to 16 years of age and were referred to the Pulmonary Function Testing Unit for the follow-up of their disease were enrolled between January 2018 and April 2019. Three groups were constituted according to the presence of asthma treatment [either on-demand (salbutamol) or continuous (inhaled corticosteroid or bronchodilator with inhaled corticosteroid or leukotriene receptor antagonist)] and SCD: SCD children without asthma treatment, SCD children with asthma treatment, and asthmatic children without SCD. For the latter group, the children had to satisfy the GINA criteria of asthma (diagnosis of asthma made by one of the hospital pediatric pulmonologist plus reversible airflow limitation in the past pulmonary function tests in the unit). Since multiple phenotypes of asthma may exist in SCD (at least asthma-like symptoms and typical asthma), all the patients underwent exhaled nitric oxide (NO), characterizing atopy (Mahut et al., 2009). SCD children were characterized as previously described, thanks to a database as part of standard patient management, VOC was defined as severe enough to require hospitalization, and ACS was defined as new pulmonary infiltrate with pain, fever, and/or hypoxemia (Sommet et al., 2016). One physician (HEJ) served as chart abstractor. An additional inclusion criterion for all children receiving anti-asthma treatment was the withdrawal of the beta-agonist before lung function tests (short-acting beta_2_-agonist >4 h and long-acting beta_2_-agonist >15 h). The sole exclusion criterion was the failure of the HRV analysis. This study was approved by an Ethics Committee (CPP SUD-EST II, 2017-17-AM1) and the database of collected data was declared to the French regulatory agency (CNIL). The subjects and their parents gave their informed consent. The Assistance Publique–Hôpitaux de Paris (DRCI) was the sponsor of the study (DrepaSympa study-K170302).

### Pulmonary Function Tests and HRV Analysis

The children/adolescents underwent functional tests, between 9:30 and 12:30 am (no acute consumption of food or water), in the following order:

– ECG recorded connection;
– Two static volume measurements using a dilution technique (normal breathing frequency: respiratory rate of 20–25/min): 5 min for each recording. These two measurements allowed the calculation of the static volumes as recommended (Wanger et al., 2005);
– One functional residual capacity (FRC) measurement using a dilution technique with slow-paced breathing recording at six cycles per minute [5 s of inspiration, 5 s of expiration: 5 min as previously described (Garcia-Araújo et al., 2015)] in order to study respiratory sinus arrhythmia (RSA) that is recognized as a physiological mechanism to ensure optimal ventilation-perfusion matching within the lungs (Hayano and Yuda, 2019);
– Exhaled NO measurement at multiple flow rates to calculate alveolar NO concentration (C_alv,NO_) and maximal bronchial NO flux (J’_aw,NO_) as previously described (Mahut et al., 2004). J’_aw,NO_ has been correlated to both subepithelial eosinophilic infiltration and reticular basement membrane thickness, two asthma characteristics (Mahut et al., 2004). FENO_0__.__05_ was also given since the modeling failure of the exhaled NO can be observed (Mahut et al., 2006);
– Baseline spirometry, accordingly to recommendations (Miller et al., 2005);
– DLCO/DLNO measurement to calculate the membrane diffusion (DM) and capillary blood volume (VC), as recommended (Zavorsky et al., 2017) (finite θ NO value), in order to characterize SCD (Lunt et al., 2018);
– Salbutamol administration via a space inhaler (400 μg) (Miller et al., 2005);
– Spirometry 15 min after salbutamol administration to calculate the bronchodilator response as compared to the baseline value;
– ECG recorder withdrawal.

Electrocardiographic recording during the pulmonary function tests was acquired with the Holter recorder SpiderView (ELA Medical, SORIN Group, Clamart, France). Analog data were edited on a SyneScope station and further exported in ASCII files. The files were then processed using the HRV analysis software 1.1 downloaded at^1^ and validated by Pichot et al. (2016).

Four 5-min periods over the approximately 1-h recording were selected:

– Two baseline measurements during the normal breathing FRC measurements that were averaged;
– One slow-paced ventilation recording during the slow-paced breathing measurement;
– One recording 10 to 15 min after salbutamol administration (before spirometry). This time point was chosen according to the results of Eryonucu et al. (2001).

From each 5 min recording time and frequency, the domain HRV variables were calculated. Time-domain variables included the mean sinus heart rate (HR), the standard deviation of the RR intervals (SDNN), the percentage of normal consecutive RR intervals differing by N50 ms (pNN-50) and the root mean of squared successive differences (RMSSD). After fast Fourier transform, the power spectrum indices were calculated as recommended (No author, 1996). The spectrum was calculated using Welch’s periodogram algorithm with a Hamming window of 256 points, an overlap of 50%, and a precision of 256 points/Hz. The total power (Ptot), very low frequencies (VLF: 0–0.04 Hz), low frequencies (LFa: 0.04–0.15 Hz), and high frequencies (HFa: 0.15–0.40 Hz) were calculated. LFa and HFa were also expressed as normalized values LFnu = 100 * LFa/(Ptot-VLF) and HFnu = 100 * HFa/(Ptot-VLF), and the LF/HF ratio was calculated.

In accordance with Billman (2013b), LF/HF = (0.50 parasympathetic + 0.25 sympathetic nerve activity)/(0.90 parasympathetic + 0.10 sympathetic nerve activity). Thus, an increase in the LF/HF ratio cannot be assumed to necessarily reflect a shift to “sympathetic dominance” and a decrease in this index as a shift to “parasympathetic dominance” (Billman, 2013b). Moreover, there is a non-linear interaction between the LF and HF that further complicates the interpretation of this ratio.

The paced ventilation response (effect of RSA) was calculated as Δ (stimulated minus baseline condition) of the normalized HF (HFnu).

Since HR variations *per se* influence HRV responses, frequency data (power spectra) were corrected by division with the corresponding mean R-R interval in seconds (Billman, 2013a), giving LHa_corrected_ and HFa_corrected_.

Lucini et al. (2018) determined that the HRV variables can be classified in three independent factors that were used: oscillatory domain (HFnu, LFnu, LF/HF ratio, and possibly ΔHFnu), amplitude domain (Ptot, HFa, and LFa) and a pulse domain (HR and mean RR). When HR fluctuations mediated by autonomic nerves are observed in HF band (>0.15 Hz), it is mediated by the cardiac vagus (Hayano and Yuda, 2019).

All pulmonary function tests and HRV indices were also recorded on a standardized abstraction form.

### Statistical Analyses

Sample size calculation. We planned to include 30 children in each group of patients based on the number of patients included in the previous studies devoted to the ANS function, involving asthmatic or SCD patients (Eryonucu et al., 2001; Pearson et al., 2005; Knight-Madden et al., 2013; Garcia-Araújo et al., 2015).

The results were expressed as median (25–75th percentiles) since most indices followed not normal distribution (HRV indices for instance). Comparisons between the baseline and stimulated conditions were performed using Wilcoxon signed-rank test. Comparisons of the continuous variables between the three groups of children were performed using the Kruskal–Wallis test, and subsequent intergroup comparisons were performed using the Mann–Whitney *U* test. Categorical variables were compared using the chi-square test. Correlations were evaluated using Pearson’s correlation coefficient. A *p* value <0.05 was deemed significant. No correction for multiple testing was done due to the pathophysiological design of the study (Rothman, 1990). All statistical analyses were performed with StatView 5.0 software (SAS Institute, Cary, NC, United States).

## Results

The HRV analysis was not obtained for one asthmatic child without SCD, leaving 89 children included. The clinical and functional characteristics of the enrolled patients are described in Table 1. SCD children with asthmatic symptoms were characterized by a more severe disease, as evidenced by the increased frequency of VOC and ACS. Among these asthmatic SCD children, six were suffering from typical atopic asthma involving several members of the family, including parents, without SCD. Thus, the typical asthma prevalence in our SCD children was 6/60 (10%, 95% CI: 2–18). Overall, the SCD children were more often treated by a short-acting bronchodilator than asthmatic non-SCD children who received ICS/LABA combination more frequently.

**TABLE 1:** Clinical characteristics of the 89 enrolled children.

**Clinical characteristics**	**Non asthmatic SCD *N* = 30**	**Asthmatic SCD *N* = 30**	**Asthma without SCD *N* = 29**	***P*value**
Age, years	12.7 [10.9; 14.5]	12.9 [10.7; 14.0]	11.4 [10.1; 12.7]	0.058
Sex, female/male	18/12	14/16	18/11	0.430
Height, cm	153 [142; 162]	152 [144; 163]	154 [142; 159]	0.878
Weight, kg	40.5 [34.0; 51.0]	39.5 [33.0; 49.0]	42.0 [38.7; 60.5]	0.214
***Asthma characteristics***				
Typical asthma, n	0	6	29	ND
GINA, 0/1/2/3/4		14/10/3/3/0	17/3/5/3/1	0.236
Controlled asthma, n		14	17	0.438
SABA, n		18	7	0.002
ICS, n		1	1	>0.999
LTRA, n		0	1	0.492
ICS/LABA, n		11	20	0.019
***SCD characteristics***				
Genotype, SS, SC, Sβ°	22/6/2	26/3/1		0.435
Deficit G6PD, n	1	1		>0.999
VOC^#^, n patients	22	27		0.181
VOC^#^, n last year	0.0 [0.0; 0.5]	1.0 [0.0; 2.0]		0.002
ACS, n patients	5	14		0.025
ACS, total number/child	0.0 [0.0; 0.0]	0.0 [0.0; 1.0]		0.018
Cerebral vasculopathy*, n	7	12		0.267
Hydroxyurea	16	20		0.429
Previous transfusion program	7	7		>0.999
Hemoglobin, g/dL	9.4 [8.4; 10.5]	8.5 [7.8; 9.0]	NA	0.054
Leucocyte count, × 10^+9^/L	7.7 [6.4; 10.2]	8.2 [7.3; 10.3]	NA	0.391
Reticulocyte count, × 10^+9^/L	188 [148; 270]	197 [147; 308]	NA	0.554
Lactic dehydrogenase, IU/L	491 [355; 621]	511 [418; 941]	NA	0.114

SABA, short-acting beta_2_-agonist; ICS, inhaled corticosteroid; LTRA, leukotriene receptor antagonist; ICS/LABA, combination of low dose of ICS and long-acting beta_2_-agonist. NA denotes Not Available. ^#^Vaso-occlusive crisis requiring hospitalization. *Cerebral vasculopathy has been defined as high stroke risk as previously done (Sommet et al., 2016).

### Pulmonary Function Tests (Table 2 and Table A1)

Asthmatic subjects, with or without SCD, depicted a mild baseline airflow limitation (reduced FEV_1_/FVC *z*-score) and a higher bronchodilator response than non-asthmatic SCD children. Increased exhaled NO values were observed in non-SCD asthmatic children when compared to SCD children, whether or not they were suffering from asthma. Bronchodilator response positively correlated with both J’_aw,NO_ (*R* = + 0.26, *p* = 0.019, *n* = 83) and FENO_0__.__05_ (*R* = + 0.22, *p* = 0.043, *n* = 89). The median DLCO values% predicted were elevated in the three groups of children, and even significantly higher in asthmatic non-SCD children as compared to SCD children (see Table A1). The VC/VA values of the SCD groups were higher than that of asthmatic non-SCD children.

**TABLE 2:** Lung functional characteristics of the 89 enrolled children.

**Functional characteristics**	**Non asthmatic SCD *N* = 30 (group 1)**	**Asthmatic SCD *N* = 30 (group 2)**	**Asthma without SCD *N* = 29 (group 3)**	***P* Value**	**Intergroup Comparisons^#^**
***Baseline lung function***					
FEV_1;_ L	2.17 [1.65; 2.64]	1.97 [1.55; 2.38]	2.06 [1.83; 2.50]	0.383	
FEV_1_, % predicted	101 [85; 106]	88 [78; 97]	100 [92; 105]	0.003	2 < 1 = 3
FEV_1_ z-score	+0.07 [−1.17; +0.46]	−0.89 [−1.65; −0.26]	+0.00 [−0.60; +0.50]	0.003	2 < 1 = 3
FVC, L	2.34 [1.94; 2.93]	2.28 [1.87; 2.75]	2.49 [2.22; 3.01]	0.334	
FVC, % predicted	99 [86; 105]	95 [84; 102]	105 [99; 118]	0.001	1 = 2 < 3
FVC, z-score	−0.08 [−1.15; +0.41]	−0.43 [−1.24; +0.16]	+0.40 [−0.05; +1.44]	0.001	1 = 2 < 3
FEV_1_/FVC	0.89 [0.86; 0.90]	0.84 [0.80; 0.86]	0.83 [0.79; 0.86]	0.001	1 > 2 = 3
FEV_1_/FVC, z-score	−0.00 [−0.44; +0.53]	−0.71 [−1.25; −0.29]	−0.99 [−1.57; −0.25]	0.001	1 > 2 = 3
Exhaled NO*					
C_alv,NO_, ppb (*n* = 83)	3.2 [2.3; 5.3]	3.4 [1.6; 5.4]	7.1 [3.3; 11.4]	0.003	1 = 2 < 3
J’_aw_,_N0_, nL/min (*n* = 83)	22 [16; 35]	16 [9; 46]	57 [24; 104]	0.001	1 = 2 < 3
FENO_005_,Ppb (*n* = 89)	10.3 [6.9; 15.0]	8.0 [5.4; 18.8]	20.8 [13.8; 34.5]	0.001	1 = 2 < 3
***Post-bronchodilator***					
FEV_1_ L	2.18 [1.73; 2.69]	2.07 [1.70; 2.45]	2.19 [1.92; 2.64]	0.425	
FEV_1_, % predicted	104 [89; 109]	94 [87; 103]	108 [102; 117]	0.001	1 = 2 < 3
FEV_1_, z-score	+0.30 [−0.86; +0.64]	−0.49 [−1.03; +0.24]	+0.63 [+0.15; +1.33]	0.001	1 = 2 < 3
Bronchodilator response, %	+3 [−1; +5]	+4 [+2; +9]	+7 [+3; +12]	0.004	1 < 2 = 3
FVC, L	2.40 [1.97; 2.85]	2.29 [1.98; 2.74]	2.51 [2.27; 3.15]	0.277	
FVC, % predicted	100 [86; 107]	96 [86; 104]	110 [102; 117]	0.001	1 = 2 < 3
FVC, z-score	+0.02 [−1.09; +0.51]	−0.34 [−1.11; +0.32]	+0.77 [+0.15; +1.33]	0.001	1 = 2 < 3
FEV_1_/FVC,	0.91 [0.88; 0.92]	0.88 [0.84; 0.91]	0.86 [0.83; 0.90]	0.005	1 > 2 = 3
FEV_1_/FVC, z-score	+0.47 [−0.25; +0.73]	+0.05 [−0.52; +0.36]	−0.36 [−0.74; +0.16]	0.008	1 > 2 = 3

*Alveolar NO concentration (C_*alv,NO*_) and maximum NO flux from bronchial tree (J’_*aw,NO*_) were not obtained in six subjects in whom the linearity of the relationship between the exhaled NO flux and expiratory rate was not observed (failure of the model) (Mahut et al., 2006). ^#^*Post hoc* two-group comparisons were performed using the Mann–Whitney *U* test or the chi-square test, as appropriate (see section “Materials and Methods”). For additional lung function tests, see Table A1.

### Baseline HRV Variability (Table 3)

Sickle cell disease children were characterized by a reduced HRV (SDNN, Ptot for instance) that was related to the reduced values of both HFa and LFa of similar degrees since the HFnu and LFnu and LF/HF ratio were similar. Thus, only the amplitude domain was decreased. HFnu, and logically LFnu, significantly correlated with the FEV_1_ *z*-score (*R* = 0.22, *p* = 0.034, *R* = −0.23, *p* = 0.030; respectively). No other significant correlation was observed between the HRV parameters and pulmonary function test parameters, which were evaluated since airflow limitation could have been related to enhanced parasympathetic tone.

**TABLE 3:** Baseline heart rate variability characteristics of the 89 enrolled children.

**Functional characteristics**	**Non asthmatic SCD**	**Asthmatic SCD**	**Asthma without SCD**	***P* value**	**Intergroup**
**Baseline HRV**	***N* = 30 (group 1)**	***N* = 30 (group 2)**	***N* = 29 (group 3)**		**comparisons**
***Pulse domain***					
RR, ms	680 [619; 714]	682 [649; 736]	685 [639; 747]	0.696	
HR, beats/min	89 [85; 97]	89 [82; 93]	88 [82; 95]	0.755	
***Amplitude domain***					
pNN50, %	26 [13; 41]	18 [7; 33]	29 [15; 39]	0.118	
SDNN, ms	60 [48; 74]	54 [40; 83]	75 [61; 97]	0.006	1 = 2 < 3
RMSSD, ms	45 [32; 63]	40 [27; 66]	53 [37; 75]	0.128	
Ptot, ms^2^	3454 [1824; 5017]	2264 [1187; 4947]	5396 [3246; 7397]	0.007	1 = 2 < 3
VLF, ms^2^	1006 [592; 2209]	779 [387; 1588]	1686 [1124; 3146]	0.012	1 = 2 < 3
LFa, ms^2^	857 [449; 1414]	593 [366; 1430]	1318 [669; 1925]	0.028	1 = 2 < 3
HFa, ms^2^	924 [446; 1453]	661 [327; 1145]	1414 [692; 2288]	0.010	1 = 2 < 3
***Oscillatory domain***					
LFnu, %	44 [35; 50]	48 [35; 55]	45 [30; 55]	0.760	
HFnu, %	39 [31; 50]	41 [30; 48]	41 [32; 51]	0.672	
LF/HF ratio	1.19 [0.94; 1.64]	1.16 [0.83; 1.91]	1.19 [0.61; 1.80]	0.686	

### HRV Variability in Stimulated Conditions (Paced Ventilation, Salbutamol)

In the whole population (*n* = 89), as compared with the baseline, paced ventilation was associated with a significant increase in Ptot (6418 ms^2^ [3384; 10294] versus 3497 [1941; 6422], *p* < 0.001) that was related to an increase in LFa (3743 ms^2^ [1401; 7460] versus 875 [456; 1689], *p* < 0.001). Both HFa (*p* = 0.088) and HR were not modified (88 beats/min [83; 95] versus 89 [80; 97], *n* = 89; *p* = 0.359). The LH/HF ratio significantly increased with paced ventilation (5.5 [1.5; 11.3] versus 1.17 [0.79; 1.80], *p* < 0.001). Thus, both amplitude and oscillatory domains were modified. Additionally, as compared to baseline, HFa under paced ventilation significantly decreased in non-SCD asthmatic children (*p* < 0.001, *n* = 29) while it did not decrease in SCD children (*p* = 0.497, *n* = 60), explaining the trend evidenced in the whole population (*n* = 89).

**FIGURE 1:**
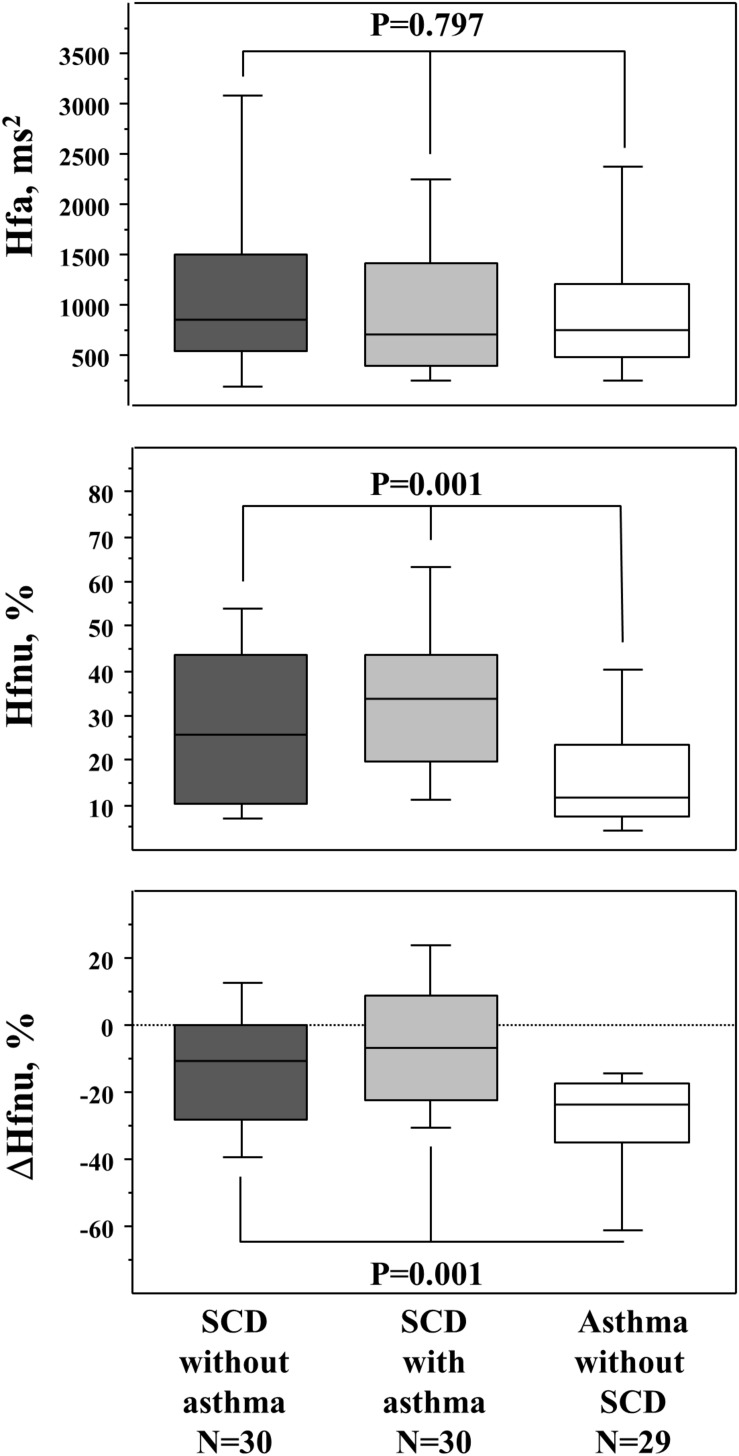
Response to paced ventilation (respiratory sinus arrhythmia evaluation). The upper panel is the HFa (raw values of HF), the middle panel is the normalized HF (Hfnu), and the lower panel is the ΔHFnu (stimulated minus baseline condition). *p* values are those of the Kruskal–Wallis test between the three groups. The Mann–Whitney *U* test further demonstrated that the two SCD groups differed from the asthmatic non-SCD group in the middle and lower panels (data not shown). Box and whisker plots show median, 25 and 75th percentiles, and 10 and 90th percentiles.

**FIGURE 2:**
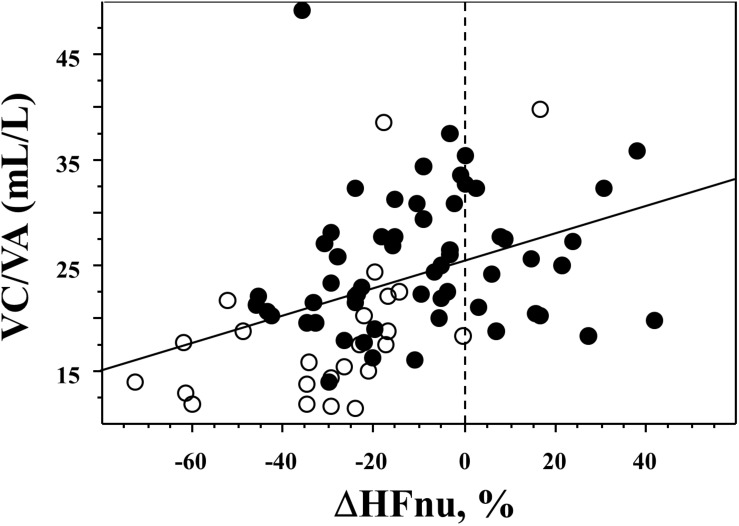
Relationship between the effect of paced ventilation and VC/VA. A significant correlation was evidenced between VC/VA and ΔHFnu: *R* = 0.406, *p* = 0.0002. Black circles are SDC children while open circles are asthmatic non-SCD children.

The response to paced ventilation differed among the three groups and is described in Figure 1; asthmatic non-SCD children were characterized by lower HFnu during paced ventilation when compared to SCD children while their HFa values were similar. Asthmatic non-SCD children exhibited a higher decrease in ΔHFnu. This decrease correlated with VC/VA (Figure 2). HFnu during paced ventilation also correlated with VC/VA (*R* = 0.373, *p* < 0.001).

In the whole population (*n* = 89), as compared with baseline, salbutamol administration was associated with a significant increase in HR (95 beats/min [92; 102] versus 88 [83; 95], *p* < 0.001), which was even higher in SCD children when compared to asthmatic non-SCD children: 97 beats/min [93; 105] versus 94 [87; 95] (*p* = 0.024). Ptot significantly decreased after salbutamol when compared to the baseline condition (2754 ms^2^ [1583; 4882] versus 3497 [1941; 6422], *p* = 0.014), which was mainly related to a significant decrease in HFa (503 ms^2^ [233; 937] versus 943 [435; 1710], *p* < 0.001). This decrease remained significant even correcting for the HR modification: HFa_corrected_, salbutamol = 831 ms^2^/s [370; 1392] versus baseline = 1410 ms^2^/s [697; 2236], *p* < 0.001. Salbutamol administration was associated with a significant increase in the LF/HF ratio (1.57 [0.98; 2.58] versus 1.17 [0.79; 1.80], *p* = 0.005). Thus, the three domains of HRV were modified.

**FIGURE 3:**
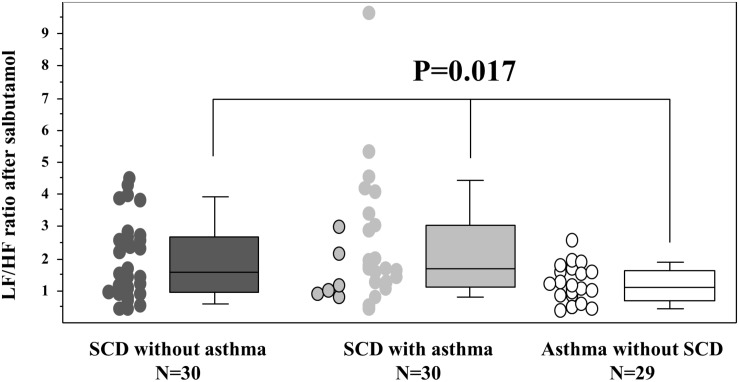
Response to salbutamol stimulation. Sympathetic stimulation was obtained after 400 μg of salbutamol administration (10 min after inhalation). The six SCD patients with typical asthma are individualized in the SCD asthma group as circles on the left side of the group with the black contour line. The *p* value is that of the Kruskal–Wallis test between the three groups. The Mann–Whitney *U* tests further demonstrated that the two SCD groups differed from the asthmatic non-SCD group (data not shown). The box and whisker plots show median, 25 and 75th percentiles, and 10 and 90th percentiles.

The response to salbutamol also differed between the three groups since asthmatic non-SCD children depicted a lower LF/HF ratio when compared to SCD children (Figure 3) and since they also depicted a higher HFnu (36% [29; 43]) versus the two SCD groups (non-asthmatic: 29% [20; 42] and asthmatic: 29% [19; 40]; *p* = 0.046).

### HRV Variability in Subgroups of SCD Children

Overall, there was no difference among all the HRV parameters between the two groups of SCD children. It has previously been emphasized that ANS dysfunction could be a marker of SCD severity; we therefore assessed ANS functions in SCD children with (*n* = 49) and without (*n* = 11) hospitalization for VOC, demonstrating that HFnu and ΔHFnu under paced ventilation were significantly different in children without any hospitalization for VOC (15% [7; 24] and −23% [−32; −8], respectively) when compared to those with (39% [19; 45] and −6% [−24; +6], respectively; *p* = 0.011 and *p* = 0.030). As previously done by Knight-Madden et al. (2013), we compared SCD children with and without history of a ACS showing that children who already had an ACS, when compared to those without a previous ACS, were characterized by lower values of both baseline HFa (404 ms^2^ [228; 895] versus 931 ms^2^ [601; 1345], respectively; *p* = 0.046) and pNN50 (13% [6;25] versus 28% [12; 41], respectively; *p* = 0.029), the latter being correlated with the number of ACS events (*R* = −0.29, *p* = 0.023).

Genotype is also a marker of severity; we compared HRV indices in severe genotypes (SS and β°, *n* = 51) as compared to a less severe genotype (SC, *n* = 9), demonstrating the absence of difference for any index.

## Discussion

Our cross-sectional pathophysiological study demonstrates that SCD children are characterized by a decreased HRV in the resting condition that further decreased upon salbutamol administration. Our working hypothesis was that either asthma diagnosis or bronchodilator treatment might aggravate SCD via their modulating effect on ANS. Our results argue for the latter hypothesis since ANS functions were similar in the two groups of SCD children while that with asthma had more vaso-occlusive events. As a consequence, these events may have been precipitated by salbutamol administration, which warrants further clinical demonstration.

### Representativeness of the Three Groups

Sickle cell disease children with asthma-like symptoms were characterized by some degree of airflow limitation and bronchodilator response when compared to non-asthmatic SCD children, explaining the prescription of asthma treatment that was mainly SABA for a cough or wheezing episodes. SCD children with asthma depicted a more severe disease according to the incidence of VOC and ACS. SCD patients with a diagnosis of asthma did not exhibit increased exhaled NO values, as previously demonstrated (Delclaux et al., 2005; Cohen et al., 2016). Asthmatic non-SCD children were characterized by an increase in exhaled NO that explained the relationship between bronchodilator response and exhaled NO, as previously evidenced (Mahut et al., 2010). They also demonstrated an increased DLCO% predicted as previously evidenced in asthmatic patients (Stewart, 1988), while their VC/VA values were lower than those of SCD children, as expected (Delclaux et al., 2005). Asthmatic SCD and asthmatic non-SCD children were clearly differentiated from a pathophysiological point of view, even if a restricted number of SCD children had typical asthma. Overall, despite the small sample of patients, every single group may seem representative of each disease state previously described.

### Representativeness of HRV

The interpretation of HRV results is a challenging issue (Billman, 2013b; Lucini et al., 2018). The first issue deals with the results of stimulated conditions when compared to the baseline condition. Stimulation by paced ventilation induced an increase in the HRV (increase in Ptot) that was related to LFa that resulted in an increase in the LF/HF ratio as previously evidenced in asthmatic and control subjects using a similar breathing pattern (Anderson et al., 2009; Garcia-Araújo et al., 2015). Stimulation by salbutamol administration induced an increase in HR, a decrease in Ptot and HFa, and an increase in LF/HF ratio as previously demonstrated in asthmatic children (Jartti et al., 1997). As a consequence, our stimulated conditions reproduced the findings of previous authors in asthmatic patients. Furthermore, the correlation between FEV_1_ and both LHnu and HFnu has previously been evidenced in both asthmatic and healthy subjects (Garcia-Araújo et al., 2015).

#### HRV Interpretation

In the baseline condition, SCD children were characterized by a frank reduction in HRV with a similar LF/HF ratio than asthmatic non-SCD children, suggesting an autonomic dysfunction in the form of impaired parasympathetic tone and sympathetic overactivity, demonstrated by previous authors using continuous non-invasive blood pressure and peripheral vascular resistance (Chalacheva et al., 2015). Interestingly, Chalacheva et al. (2015) recently demonstrated that low parasympathetic activity at baseline dramatically increased the probability of belonging to a peripheral vasoconstriction endotype in SCD subjects, even after adjusting for hemoglobin level, suggesting a characteristic ANS dysfunction that is independent of anemia (Chalacheva et al., 2019).

Paced ventilation induced an increase in Ptot, LFa, and LF/HF ratio that was expected (Hayano and Yuda, 2019). Whereas HFa decreased in asthmatic children as usually evidenced in healthy subjects, this decrease was not observed in SCD children. RSA ensures optimal ventilation-perfusion matching within the lungs (Hayano and Yuda, 2019), which is defective in SCD patients (Pianosi et al., 1991) who are characterized by an increased lung capillary blood volume (Delclaux et al., 2005). We show that the increased VC/VA of SCD children is associated with lesser effect of RSA; thus, an already recruited vascular bed is associated with the loss of RSA effect, which may participate to the mild degree of hypoxemia in SCD patients (Delclaux et al., 2005). These effects of respiratory parameters on the RSA operate independently of the level of cardiac vagal activity (Hayano and Yuda, 2019). When respiration frequency is <0.15 Hz, RSA becomes a part of the LF component, as observed in our study, and the association between HF component and cardiac vagal function is lost (Hayano and Yuda, 2019). Asthmatic non-SCD children were characterized by a more frank decrease in HF as compared to SCD children. Even though the ANS cannot transfer HRV >0.15 Hz, it may restrict the magnitude of cardiac vagal modulations of heart rate (Hayano and Yuda, 2019), which may explain the observed effects. Thus, the different effects of paced ventilation in SCD and non-SCD children may be explained by the increased sympathetic modulation in SCD.

More importantly for our study question, the two groups of SCD children were similar, suggesting that the diagnosis of asthma *per se* did not modify ANS functions. Since the SCD children with asthma-like symptoms were characterized by an increased prevalence of both VOC and ACS, one may, therefore, hypothesize that asthma treatment could have been involved in vaso-occlusive events. Along this line, salbutamol administration in SCD children further modified the ANS balance toward a deregulated profile similar to that observed during VOC. Charlot et al. (2017) showed that HFa decreased during VOC compared to the steady state, suggesting parasympathetic withdrawal that certainly caused the dominance of the sympathetic activity over the parasympathetic activity, as reflected by the increase in LF/HF during VOC. Finally, we show that markers of SCD severity (hospitalization for VOC or occurrence of ACS) were associated with ANS modifications that may suggest parasympathetic withdrawal, as previously evidenced for ACS (Knight-Madden et al., 2013).

The baseline modifications of HRV could be related to beta-adrenergic stimulation, which has been shown to result in a significant decrease in time domain measures of HRV (Ahmed et al., 1994). Epinephrine has also been shown to promote red blood cell adhesion and vaso-occlusion in SCD (Zennadi et al., 2007). Moreover, genetic variation in the beta-1 (Ashley-Koch et al., 2008) and beta-2 (Jhun et al., 2019) adrenergic receptors has been shown to influence pulmonary hypertension prevalence and chronic pain severity in SCD. Thus, sympathetic nervous system inhibition may be a therapeutic approach in SCD.

For years, the scientific community has worked under the paradigm that the asthma exacerbation, not the treatment, is the likely trigger for VOC. This made sense because asthma exacerbation limits airflow and oxygenation that would worsen sickling. Nevertheless, Sangkatumvong et al. (2011) have shown that desaturation did not induce change in microvascular perfusion in SCD patients. Therefore, other mechanisms had to be found to justify this high risk of VOC, which was the subject of this study.

### Limitations of the Study

Our study has limitations due to its design. We did not include healthy control children. Nevertheless, our objective was to assess whether the pathophysiology of asthmatic SCD was similar to that of asthmatic without SCD since one may have initially hypothesized that asthmatic SCD would depict similar findings than asthmatic without SCD when compared to SCD children without asthma, justifying their severity and our design. Furthermore, ANS comparisons have previously been obtained between both asthmatic and healthy subjects and between SCD patients and healthy subjects, showing that baseline HRV is similar in asthmatic patients and healthy subjects (Nebor et al., 2011; Garcia-Araújo et al., 2015). Importantly, our data have been obtained in baseline stable condition, which is probably not representative of the setting leading to salbutamol administration, i.e., asthma-like symptoms indicating SCD worsening. Finally, our results do not demonstrate the causality relationship between salbutamol administration and increased vaso-occlusive events in SCD children, which remains to be further evaluated in a prospective design.

In conclusion, these data suggest that SCD children have altered autonomic activity that is worsened by salbutamol administration.

## Data Availability Statement

The datasets generated for this study are available on request to the corresponding author.

## Ethics Statement

The studies involving human participants were reviewed and approved by the Ethics Committee, Comité de Proctection des Personnes hosted by Hospices Civils de Lyon (CPP SUD-EST II, 2017-17-AM1). Written informed consent to participate in this study was provided by the participants’ legal guardian/next of kin.

## Author Contributions

PB, MB, CP, and CD conceived the study. PB, ID, and CD design the study. HE, NM, LH, and CP acquired the data. HE, PB, and CD analyzed the data. ID, CP, NM, LH, MB, PB, and CD interpreted the data. All authors drafted the manuscript, critically revised the manuscript for important intellectual content, approved the final manuscript for publication, and agreed to be accountable for all aspects of the work in ensuring that questions related to the accuracy or integrity of any part of the work are appropriately investigated and resolved. CD had full access to all the data in the study and that he takes responsibility for the integrity of the data and the accuracy of the data analysis, including and especially any adverse effects.

## Conflict of Interest

The authors declare that the research was conducted in the absence of any commercial or financial relationships that could be construed as a potential conflict of interest.

## Footnotes

1 httpse://anslabtools.univ-st-etienne.fr.

## Appendix

**TABLE A1:** Static lung volumes and lung diffusion of the 89 enrolled children.

**Functional characteristics**	**Non asthmatic SCD**	**Asthmatic SCD**	**Asthma without SCD**	**P value**	**Intergroup**
	***N* = 30 (group 1)**	***N* = 30 (group 2)**	***N* = 29 (group 3)**		**comparisons^#^**
***Baseline lung function***					
TLC, L	3.14 [2.49; 3.72]	3.05 [2.67; 3.55]	3.32 [2.56; 3.79]	0.899	
TLC, % predicted	100 [90; 107]	95 [89; 102]	101 [90; 108]	0.322	
FRC, L	1.38 [1.10; 1.78]	1.46 [1.19; 1.70]	1.45 [1.07; 1.82]	0.982	
FRC, % predicted	95 [82; 114]	95 [83; 111]	97 [85; 115]	0.906	
RV, L	0.87 [0.66; 1.17]	0.89 [0.82; 1.15]	0.81 [0.56; 1.01]	0.207	
RV, % predicted	107 [83; 134]	106 [90; 127]	90 [70; 116]	0.145	
VA, L	3.14 [2.77; 3.82]	3.06 [2.64; 3.76]	3.32 [2.92; 4.07]	0.342	
VA, % predicted	99 [88; 108]	101 [84; 111]	109 [102; 123]	0.013	1 = 2 < 3
DLCO, mL/mmHg/min	18.3 [16.6; 22.9]	18.0 [14.5; 21.7]	22.0 [19.1; 24.7]	0.010	1 = 2 < 3
DLCO, % predicted	112 [98; 138]	112 [90; 124]	141 [122; 151]	0.001	1 = 2 < 3
DMco/VA, mL/mmHg/min/L	19.6 [17.5; 22.1]	18.2 [16.6; 19.3]	24.8 [21.7; 28.7]	0.001	1 = 2 < 3
VC/VA, mL/L	22.0 [20.0; 27.8]	26.5 [22.2; 29.4]	17.1 [13.3; 20.9]	0.001	1 = 2 > 3

*VA denotes alveolar volume. ^#^*Post hoc* two-group comparisons were performed using the Mann–Whitney *U* test or the chi-square test, as appropriate (see section “Materials and Methods”).*
